# Investigation of Physical Activity Levels in the Population of Switzerland: Association With Lifestyle and Sociodemographic Factors

**DOI:** 10.3389/ijph.2025.1608010

**Published:** 2025-03-11

**Authors:** Florence D. Berger, Flurina Suter, Sabine Rohrmann

**Affiliations:** Division of Chronic Disease Epidemiology, Epidemiology, Biostatistics and Prevention Institute (EBPI), University of Zurich, Zurich, Switzerland

**Keywords:** physical activity, healthy lifestyle factors, sociodemographic factors, menuCH study, ordinal logistic regression

## Abstract

**Objective:**

The aim of the present study was to investigate physical activity levels of the population of Switzerland and the association of lifestyle and sociodemographic factors with physical activity levels.

**Methods:**

The association of physical activity with lifestyle and sociodemographic factors was analyzed by fitting ordinal logistic regression models, using the data of 2057 participants from the National Nutrition Survey menuCH.

**Results:**

The physical activity level of the population of Switzerland was high: less than 10% of the participants are not physically active. Factors associated with lower physical activity levels, were the sociodemographic variables, middle age [age 30–44: odds ratio = 0.53 (95% confidence interval 0.37, 0.77) and age 45–59: 0.60 (0.41, 0.89)] and higher education [tertiary level: 0.50 (0.29, 0.86)] as well as the lifestyle factors higher body mass index [obesity: 0.64 (0.45, 0.90)] and poor self-reported health status [0.68 (0.50, 0.93)].

**Conclusion:**

To improve the physical activity level of the population of Switzerland tailored public health strategies are required that address specific groups, such as individuals in the middle age group or obese individuals.

## Introduction

Non-communicable diseases (NCD) rank prominently among the leading causes of global mortality, accounting for 71% of overall deaths [1]. The majority of all NCD deaths are caused by cardiovascular diseases (CVD, 44%), followed by cancer (22%), chronic respiratory diseases (9%), and diabetes (4%) [2]. For more than 10 years, CVD and cancer have been the first and second leading cause of death in Switzerland [3, 4].

NCD are caused by genetic, physiological, environmental, and behavioral factors, such as unhealthy diets, physical inactivity, smoking, and alcohol consumption [5–7]. Among these behavioral risk factors, physical inactivity is the fourth leading risk factor contributing to global mortality [8]. Previous studies have shown that risk factors for NCD often co-occur [9–11]. Physical activity (PA) has been linked to decreased smoking [12] and healthier eating [13, 14] but is associated with higher alcohol consumption [15–17]. Adopting a holistic healthy lifestyle such as refraining from smoking, engaging in moderate to no alcohol consumption, following a healthy diet, and maintaining a healthy body weight is therefore crucial to prevent NCD.

Engaging in PA has not only protective effects on several NCD, for example on preventing obesity and cardiovascular diseases [18]. Several guidelines, including those of the World Health Organization (WHO), recommend meeting the goal of 150 min of moderate to vigorous PA per week [19, 20]. However, there is no linear relationship of health benefits and PA [21]. An umbrella review revealed that the most health benefits are achieved when going from an inactive state into an active state, irrespective of the amount of PA per week [22].

Around the early 2000s, over 30% of the world’s population did not meet the WHO recommendations on PA [23]. In Switzerland, an increase in physically active people has been observed in the last 50 years, with 30% of the population of Switzerland being physically inactive in 1978 to only 18% in 2020 [24]. The number of people in Switzerland being physically active several times per week increased from 22% in 1978 to 57% in 2020 [24]. In 2022, a decrease in physical inactivity has been observed in Switzerland compared to 2020 mainly attributable to the restrictions (home-office, wearing of masks during indoor sport etc.) during the Coronavirus Disease 2019 pandemic [24].

Differences in PA levels were observed worldwide in different demographic and sociodemographic groups e.g., females compared to males, older adults compared to younger, and people of low sociodemographic status compared to those of high sociodemographic status [25]. Especially high-income compared to low-income countries have lower PA levels, which can mainly be explained by the motorized transportation [26].

It is crucial to understand the sociodemographic factors that impact an individual’s commitment to maintain a physically active lifestyle and thereby reducing their risk for NCD. Healthy lifestyle factors in combination with sociodemographic factors have been investigated to understand the pattern of PA [26, 27]. To the best of our knowledge, further factors such as the information on dietary intake, self-reported general health status, or if the individuals follow a weight-loss diet, were only assessed in studies from the UK [28] and the US [29] but none from Switzerland [30, 31]. To fill this gap in knowledge, our study used population-based, representative Swiss data to investigate the association of PA with healthy lifestyle and sociodemographic factors.

## Methods

The Strengthening the Reporting of Observational Studies in Epidemiology (STROBE) guidelines were followed for the current study [32].

### Study Design and Participants of menuCH Survey

Between January 2014 and February 2015, the cross-sectional Swiss National Nutrition Survey, menuCH, was conducted in ten centers across Switzerland [30, 31]. Two 24-h dietary recalls (24HDR) were conducted, along with one self-administered questionnaire that included sociodemographic, dietary, and lifestyle variables as well as details about PA [31]. The target sample of the menuCH study covered the seven major regions of Switzerland (Lake Geneva, Midlands, Northwest, Zurich, Eastern, Central, and Southern Switzerland), the three main language regions (CH-German, CH-French, CH-Italian), five age categories (18–29, 30–39, 40–49, 50–64, and 65–75 years old), and both sexes (male, female) and consisted of 4,627,878 residents. From the source study sample of 13,606 individuals 5,496 eligible residents were successfully contacted by mail and phone. A total of 2086 individuals agreed to schedule an interview. Among them, 2057 completed two 24HDR, while the remaining 29 completed only one. The 2057 individuals with two complete 24HDR were included in the current study [33].

### Physical Activity Assessment in menuCH

Data on PA was collected using the short-form International Physical Activity Questionnaire (IPAQ). The IPAQ was developed by an International Consensus Group in the late 1990s. It is a standardized tool to measure PA levels through a self-reported questionnaire with 7 questions to measure the amount and intensity of PA as well as the sitting time over the last 7 days. The IPAQ assesses PA during leisure, occupational, domestic, and commuting time, but does not distinguish between the different domains. PA is measured using the Metabolic Equivalent of Task (MET), which is a unit to measure the amount of energy expenditure of PA (walking = 3.3 METs, moderate PA = 4.0 METs, vigorous PA = 8.0 METs) [34, 35]. In our study, the participants were classified into three levels (high, moderate, and low). Participants in the high and moderate levels meet the recommended level of PA according to WHO [20]. The category high included participants who met any of the following criteria: vigorous-intensity activity on at least 3 days achieving a minimum of at least 1500 MET-minutes/ week or 7 or more days of a combination of walking, moderate-intensity, or vigorous-intensity activities reaching a minimum of at least 3000 MET-minutes/week. The category moderate included participants meeting any of the following criteria: 3 or more days with at least 20 min of vigorous-intensity activity, 5 or more days of moderate-intensity or walking of at least 30 min per day, 5 or more days of any combination of walking, moderate-intensity, or vigorous-intensity activities accumulating a minimum of at least 600 MET-minutes/week. The category low included participant who did not meet the criteria for moderate or high PA levels [34].

### Lifestyle and Sociodemographic Factors

The menuCH study included data on the participants’ lifestyle and sociodemographic factors, which were collected with a self-administered questionnaire [33]. The following lifestyle factors were included in our study: Body mass index [(BMI); calculated using the participants’ body weight and height and classified according to WHO definitions: underweight (BMI <18.5 kg/m^2^), normal weight (18.5 kg/m^2^ ≤ BMI <25.0 kg/m^2^), overweight (25.0 kg/m^2^ ≤ BMI <30.0 kg/m^2^), obese (BMI ≥30 kg/m^2^)], smoking habits (never, former, current), currently following a weight-loss diet (yes, no), and self-reported health status (good, medium-bad). The following sociodemographic variables were used in our study: sex (male, female), age group (divided in 4 categories: 18–29, 30–44, 45–59, and 60–75 years old), Swiss language region (CH-German, CH-French, CH-Italian), education level (primary, secondary, tertiary), and civil status (married, divorced, single, other).

### Dietary Assessment in menuCH

Two 24HDR interviews were performed. The first interview was conducted face-to-face and 2 weeks later the second interview was conducted, which was done over the phone. Both interviews were performed by trained dietitians [36]. The food items reported in the interview were grouped in 19 categories using the trilingual Swiss version (0.2014.02.27) of the automated software GloboDiet^®^ (GD, formerly EPIC-Soft^®^, IARC; Lyon, France [37, 38], adapted by the Federal Food Safety and Veterinary Office, Berne, Switzerland). The quantification of the consumed amounts was done with a book including 119 series of six graduated portion-size pictures and 60 actuals household measures, which were presented to the participants [39].

The menuCH participants were categorized into three alcohol consumption categories (none, moderate, heavy) based on recommended intake thresholds given by the Swiss Federal Office of Public Health [40]. A male participant was classified as a heavy drinker if his daily alcohol consumption exceeded 24 g of pure alcohol, and a female participant was classified as a heavy drinker if her daily alcohol consumption exceeded 12 g of pure alcohol. A male participant consuming 24 g or less of pure alcohol per day and a female participant consuming 12 g or less of pure alcohol per day were classified as moderate drinkers [41]. Non-alcohol-consumer were those participants who declared in the self-administered questionnaire to avoid drinking alcohol and did not report any alcohol consumption during either 24HDR.

The Alternative Healthy Eating Index (AHEI) score was calculated based on ten components (0–10 points per component), namely vegetables, fruits, whole grains, sugar sweetened beverages, nuts, meat, trans fat, long chain omega-3 fatty acids, polyunsaturated fatty acids, sodium, and alcohol. Higher AHEI scores indicate a higher diet quality [42]. The AHEI score is calculated for each 24HDR and the average of both AHEI scores was determined. The alcohol component was excluded from the AHEI score used in our analysis, as alcohol was considered as a separate exposure variable.

### Statistical Analysis

The menuCH study sample was weighted based on sex, age, marital status, major living region, nationality, household size, weekday and season of the 24HDR day to ensure having a representative sample of the population of Switzerland. The weighting strategy is described in detail in the open survey data repository [43].

Descriptive statistics [absolute numbers, percentages, median, and interquartile range (IQR)] were used to characterize the study population.

Multivariate imputation by chained equations (MICE) was used to impute missing values for PA level (n = 524), education level (n = 3), sitting time (n = 585), currently being on a diet (n = 4), self-reported health status (n = 4), civil status (n = 3), and smoking category (n = 4) [44]. Rubin’s rule was used to pool the results of the 25 imputed data sets [45].

Ordinal regression models were fitted to investigate the association between PA level and sociodemographic and lifestyle factors. The dependent variable, PA level, was defined as an ordinal outcome because it has a natural order (low < moderate < high). Therefore, an ordinal logistic regression approach was chosen, adjusted for sex, age group, Swiss-language region, education level, civil status, nationality, smoking, alcohol intake, BMI, health status, and currently on a diet. All confounders were chosen a priory.

Separate models were fitted for males and females to explore potential sex-based differences in the associations between PA levels and the sociodemographic and lifestyle factors.

To assess the goodness-of-fit of the model, the Hosmer-Lemeshow test and the Pulkstenis-Robinson test were conducted with each 25 imputed data sets. Subsequently, the median estimates were determined. The Hosmer-Lemeshow test groups the observations based on deciles of predicted probabilities for the outcome variables and compares the observed and expected frequencies within each group using a Chi-squared statistic. The Pulkstenis-Robinson test groups observations into homogenous groups based on predicted probabilities for the outcome variables and compares observed and expected frequencies within each group. Chi-squared test and deviations tests are used to provide p-values to assess model fit [46].

For the sensitivity analysis, the original ordinal regression model was additionally adjusted for income and/or sitting time as explanatory variables. The first model included income as additional explanatory variable, the second model sitting time, and the third model both, income and sitting time, to explore the effect on PA levels.

The R programming language (version 4.1.2) was used to conduct all analyses. The following R packages were used for weighting (stats), ordinal logistic regression (MASS), multivariate imputation by chained equations (mice) and goodness-of-fit tests (generalhoslem) [47]. The statistical significance level was set to 0.05 for all analyses.

## Results

### Descriptive Results

Baseline characteristics of the menuCH participants stratified by PA level are shown in Table 1. The largest group was the high PA group with 827 (40.2%) individuals, followed by the moderate PA group with 487 (23.7%) individuals, and the smallest group was the low PA group with 219 (10.7%) individuals. 524 (25.5%) individuals did not provide complete information on PA and therefore the PA level could not be determined. 63.9% of all participants (participants with a moderate and high PA level) met the PA recommendations, which is a minimum of 150 min of moderate PA. Participants aged 30 to 59 were more likely to be in the low PA group, compared to the younger (aged 18–29) and older (aged 60–76) participants. German- and French-speaking participants were more likely to be in the low PA group compared to the Italian-speaking participants, as well as obese and overweight participants compared to the underweight and normal weight participants. No trend in alcohol intake, AHEI score, smoking status, civil status, or nationality among the PA levels was observed. Individuals with a low PA level had higher median daily sitting time (8 h) than individuals in the moderate (7 h) and high (5 h) PA group.

**TABLE 1:** Baseline characteristics of menuCH participants (n = 2057) stratified by physical activity group^a^ (National Nutrition Survey menuCH, Switzerland, 2014–2015).

Variable	Overall	Low	Moderate	High	NA
n	2057	219	487	827	524
Female, n (%, %*)	1,124 (54.6, 50.2)	96 (43.8, 39.6)	280 (57.5, 54.2)	422 (51.0, 48.2)	326 (62.2, 54.4)
Age (IQR)	47 (33, 59)	46 (37, 54)	45 (32, 59)	48 (32, 60)	46 (33, 59)
Age group, n (%, %*)					
18–29	400 (19.4, 18.8)	26 (11.9, 8.2)	89 (18.3, 15.5)	176 (21.3, 22.5)	109 (20.8, 20.8)
30–44	533 (25.9, 29.9)	71 (32.4, 40.1)	141 (29.0, 34.4)	183 (22.1, 25.3)	138 (26.3, 28.5)
45–59	625 (30.4, 29.6)	87 (39.7, 38.4)	138 (28.3, 28.5)	250 (30.2, 28.1)	150 (28.6, 29.3)
60–76	499 (24.3, 21.6)	35 (16.0, 13.4)	119 (24.4, 21.7)	218 (26.4, 24.1)	127 (24.2, 21.4)
Language region, n (%, %*)^b^					
German	1,341 (65.2, 68.8)	156 (71.2, 72.3)	309 (63.4, 69.0)	542 (65.5, 68.9)	334 (63.7, 66.8)
French	502 (24.4, 25.7)	36 (16.4, 20.8)	125 (25.7, 25.0)	209 (25.3, 26.1)	132 (25.2, 27.8)
Italian	214 (10.4, 5.6)	27 (12.3, 6.9)	53 (10.9, 6.0)	76 (9.2, 5.0)	58 (11.1, 5.4)
Education level, n (%, %*)					
Primary	89 (4.3, 4.6)	6 (2.7, 2.8)	16 (3.3, 3.0)	38 (4.6, 5.5)	29 (5.5, 5.7)
Secondary	968 (47.1, 43.1)	96 (43.8, 41.1)	205 (42.1, 36.2)	388 (46.9, 43.3)	279 (53.2, 50.4)
Tertiary	997 (48.5, 52.0)	117 (53.4, 56.2)	266 (54.6, 60.9)	401 (48.5, 51.2)	213 (40.6, 42.8)
NA	3 (0.1, 0.3)	0 (0.0, 0.0)	0 (0.0, 0.0)	0 (0.0, 0.0)	3 (0.6, 1.1)
Nationality, n (%, %*)					
Swiss	1,492 (72.5, 60.6)	152 (69.4, 52.2)	351 (72.1, 61.1)	609 (73.6, 62.2)	380 (72.5, 61.4)
Non-Swiss	268 (13.0, 25.0)	41 (18.7, 35.8)	64 (13.1, 25.1)	99 (12.0, 22.7)	64 (12.2, 23.7)
Swiss binationals	297 (14.4, 14.3)	26 (11.9, 11.9)	72 (14.8, 13.8)	119 (14.4, 15.1)	80 (15.3, 14.8)
Civil status, n (%, %*)					
Single	634 (30.8, 30.9)	63 (28.8, 28.6)	155 (31.8, 29.9)	257 (31.1, 32.9)	159 (30.3, 29.6)
Married	1,125 (54.7, 52.5)	124 (56.6, 56.2)	269 (55.2, 55.9)	451 (54.5, 50.5)	281 (53.6, 50.9)
Divorced	223 (10.8, 11.7)	25 (11.4, 11.1)	44 (9.0, 9.2)	92 (11.1, 12.1)	62 (11.8, 13.9)
Other	72 (3.5, 4.6)	7 (3.2, 4.1)	19 (3.9, 5.0)	27 (3.3, 4.4)	19 (3.6, 4.6)
NA	3 (0.1, 0.3)	0 (0.0, 0.0)	0 (0.0, 0.0)	0 (0.0, 0.0)	3 (0.6, 1.1)
BMI (IQR)	24.3 (21.8, 27.2)	25.4 (22.7, 28.7)	24.0 (21.4, 27.4)	24.1 (21.8, 26.8)	24.3 (21.8, 27.5)
BMI group, n (%, %*)					
Underweight	51 (2.5, 2.3)	2 (0.9, 0.3)	11 (2.3, 2.4)	19 (2.3, 2.0)	19 (3.6, 3.7)
Normal	1,115 (54.2, 54.4)	100 (45.7, 47.5)	266 (54.6, 54.0)	474 (57.3, 58.9)	275 (52.5, 50.6)
Overweight	629 (30.6, 30.7)	74 (33.8, 33.2)	151 (31.0, 32.2)	255 (30.8, 29.4)	149 (28.4, 30.3)
Obese	262 (12.7, 12.5)	43 (19.6, 19.0)	59 (12.1, 11.4)	79 (9.6, 9.7)	81 (15.5, 15.4)
Currently on a diet, n (%, %*)					
No	1940 (94.3, 93.9)	207 (94.5, 94.9)	453 (93.0, 92.8)	789 (95.4, 95.7)	491 (93.7, 91.8)
Yes	113 (5.5, 5.7)	12 (5.5, 5.1)	34 (7.0, 7.2)	38 (4.6, 4.3)	29 (5.5, 7.0)
NA	4 (0.2, 0.3)	0 (0.0, 0.0)	0 (0.0, 0.0)	0 (0.0, 0.0)	4 (0.8, 1.3)
Health status, n (%, %*)					
Good	1781 (86.6, 93.9)	182 (83.1, 94.9)	420 (86.2, 92.8)	743 (89.8, 95.7)	436 (83.2, 91.8)
Medium-bad	272 (13.2, 5.7)	37 (16.9, 5.1)	67 (13.8, 7.2)	84 (10.2, 4.3)	84 (16.0, 7.0)
NA	4 (0.2, 0.3)	0 (0.0, 0.0)	0 (0.0, 0.0)	0 (0.0, 0.0)	4 (0.8, 1.3)
Alcohol intake, n (%, %*)					
None	870 (42.3, 41.0)	99 (45.2, 47.1)	188 (38.6, 35.5)	356 (43.0, 41.0)	227 (43.3, 43.4)
Moderate	613 (29.8, 29.3)	63 (28.8, 28.5)	154 (31.6, 32.2)	253 (20.6, 29.8)	143 (27.3, 26.0)
Heavy	574 (27.9, 29.8)	57 (26.0, 24.4)	145 (29.8, 32.4)	218 (26.4, 29.2)	154 (29.4, 30.6)
Smoking, n (%, %*)					
Never	914 (44.4, 42.0)	98 (44.7, 43.2)	212 (43.5, 41.1)	380 (45.9, 42.2)	224 (42.7, 40.8)
Former	688 (33.4, 35.4)	80 (36.5, 38.6)	161 (33.1, 33.7)	271 (32.8, 35.4)	176 (33.6, 35.4)
Current	451 (21.9, 22.4)	41 (18.7, 18.8)	114 (23.4, 25.2)	176 (21.3, 28.4)	120 (22.9, 25.6)
NA	4 (0.2, 0.3)	0 (0.0, 0.0)	0 (0.0, 0.0)	0 (0.0, 0.0)	4 (0.8, 1.3)
AHEI (IQR)^c^	40.1 (31.9, 48.4)	37.0 (29.8, 46.5)	41.9 (33.0, 49.6)	40.3 (32.0, 49.1)	38.9 (30.8, 47.2)
Sitting time, h (IQR)	6.0 (4.0, 9.0)	8.0 (5.0, 10.8)	7.0 (4.5, 10.0)	5.0 (3.5, 8.0)	NA

BMI, body mass index; IQR, interquartile range; AHEI, alternative healthy eating index; NA, missing; h, hours.

^a^ Categorical variables are represented as absolute number (n), unweighted percentage (%), and weighted percentage (%*). Continuous variables are expressed as weighted median and interquartile range (IQR). The weighted percentages (%*), weighted median, and weighted IQR are weighted according to the menuCH weighting strategy [43]. The weighting strategy included sex, age, marital status, major living region Switzerland, nationality, household size, weekday and season of the recall day [43].

^b^ German language region: cantons Aargau, Basel City, Basel Country, Berne, Lucerne, Zurich, and St. Gallen. French language region: cantons Jura, Neuchâtel, Vaud, and Geneva. Italian language region: canton Ticino.

^c^ The AHEI is calculated as average AHEI of the two 24-h dietary recall. It compromises of ten components, which contribute each 0 to 10 points: vegetables, fruits, whole grains, sugar sweetened beverages, nuts, meat, trans fat, long chain omega-3 fatty acids, polyunsaturated fatty acids (PUFA), and sodium. The higher the AHEI score, the healthier the participant’s diet. Alcohol is not included in the AHEI score of the current study, because it is investigated as a separate value [42].

### Ordinal Logistic Regression

The association between PA and several sociodemographic and lifestyle factors based on an ordinal logistic regression model are shown in Table 2. The findings suggested an influence of the sociodemographic factors, as well as indicating an association with the lifestyle factors BMI and self-reported health status and PA level. Sociodemographic variables, such as age and education level emerged as notable correlates of PA level. Specifically, individuals aged 30–44 and 45–59 in comparison to those aged 18–29 had lower odds [OR = 0.53, 95% CI (0.37, 0.77) and 0.60 (0.41, 0.89)] of being in a higher PA level (compared to the lower PA levels). Similarly, individuals with tertiary education in comparison to individuals with a primary education had lower odds [0.50 (0.29, 0.86)] of being in a higher PA level (compared to the lower PA levels).

**TABLE 2:** Results of the ordinal logistic regression model for sociodemographic and lifestyle factors associated with physical activity level (n = 2057). (National Nutrition Survey menuCH, Switzerland, 2014–2015).

	OR^a^	[95% CI]
Sex		
Male (ref.)	1.00	-
Female	0.93	[0.76, 1.15]
BMI		
Normal (ref.)	1.00	-
Underweight	1.18	[0.60, 2.36]
Overweight	0.84	[0.67, 1.05]
Obese	0.64	[0.45, 0.90]*
Age		
18–29 (ref.)	1.00	-
30–44	0.53	[0.37, 0.77]*
45–59	0.60	[0.41, 0.89]*
60–76	0.88	[0.56, 1.31]
Language region		
German (ref.)	1.00	-
French	1.11	[0.87, 1.42]
Italian	0.85	[0.61, 1.29]
Nationality		
Swiss (ref.)	1.00	-
Non-Swiss	0.81	[0.61, 1.09]
Swiss binationals	0.99	[0.74, 1.33]
Education		
Primary (ref.)	1.00	-
Secondary	0.58	[0.33, 1.02]
Tertiary	0.50	[0.29, 0.86]*
Health status		
Good (ref.)	1.00	-
Medium-bad	0.68	[0.50, 0.93]*
Smoking		
Never (ref.)	1.00	-
Former	1.00	[0.80, 1.26]
Current	1.01	[0.78, 1.33]
Civil status		
Single (ref.)	1.00	-
Married	1.05	[0.81, 1.36]
Divorced	1.25	[0.86, 1.83]
Other	0.89	[0.56, 1.38]
Currently on a diet		
No (ref.)	1.00	-
Yes	0.89	[0.56, 1.38]
Alcohol intake		
None (ref.)	1.00	-
Moderate	0.97	[0.76, 1.25]
Heavy	1.00	[0.78, 1.29]
AHEI score	1.00	[0.99, 1.01]

*p < .05.

BMI, body mass index; AHEI, alternative healthy eating index; OR, odds ratio; CI, confidence interval.

^a^ The OR are weighted according to the menuCH weighting strategy for sex, age, marital status, major living region Switzerland, nationality, household size, weekday and season of the recall day [43].

The other sociodemographic factors, namely sex, language region, nationality, and civil status, did not reveal evidence for an association with PA levels.

The two lifestyle factors that were significantly associated with PA levels, were BMI and self-reported health status. Obese individuals in comparison with normal weight individuals had lower odds [0.64 (0.45, 0.90)] of being in a higher PA level (compared to lower PA levels). Individuals reporting a medium to bad health status had lower odds [0.68 (0.50, 0.93)] of being in a higher PA level (compared to the lower PA levels) compared to individuals reporting a good health status. For the other lifestyle factors (smoking status, being on a diet, alcohol consumption, and AHEI score), no association with PA levels was observed.

To explore the sex-based differences in the association between PA and sociodemographic and lifestyle factors, the analysis was stratified by sex. The findings suggest that there is a difference between males and females. In the model including only females, we did not observe any statistically significant associations between sociodemographic and lifestyle factors and PA level (Supplementary Table SI 1). In the male-only model, the following associations are consistent with those in the main model: age 30–44 [0.38 (0.11, 0.69)] and 45–59 [0.43 (0.23, 0.79)] years, education [tertiary 0.45 (0.42, 0.95)], and self-reported health status [bad to medium 0.63 (0.42, 0.95)]. In contrast, BMI was no longer significantly associated with PA, but secondary education was associated [0.46 (0.21, 0.99)] (Supplementary Table SI 1).

### Goodness-Of-Fit

The Hosmer-Lemeshow and the Pulkstenis-Robinsons tests were performed to assess the goodness-of-fit of the ordinal logistic regression model. The Hosmer-Lemeshow test produced a Chi-squared statistic of 15.285 (df = 7, p = 0.033), indicating a potential lack of fit. The Pulkstenis-Robinson test yielded a median Chi-squared test statistic of 3,598.6 (df = 3,528, p = 0.200) and the deviance test resulted in a median value of 3,485.8 (df = 3,528, p = 0.690). Both, the Chi-squared test and the deviance test, indicated no significant lack of fit.

### Sensitivity Analysis

We conducted a sensitivity analysis exploring the variables income and sitting time as additional explanatory variables in the regression analysis on the association of sociodemographic and lifestyle factors with PA levels (Supplementary Table SI 2–4). The regression model additionally adjusted for income (Supplementary Table SI 2) indicated that income was not statistically significantly associated with PA. The regression model additionally adjusted for sitting time (Supplementary Table SI 3) revealed that sitting time was negatively associated with PA level, while education was no longer significantly associated with PA level. Including both, income and sitting time, as additional explanatory variables in the regression model (Supplementary Table SI 4) confirmed that sitting time remained negatively associated with PA level, while income still was not.

## Discussion

The population of Switzerland was in general physically active with the majority of the participants belonging to the moderate and high PA level. PA levels were associated with the sociodemographic factors age group and education level. Among the lifestyle factors, we observed an association of BMI group and self-reported general health status with PA level. By performing the analysis stratified by sex, we could detect a difference among the sexes. The results for the males are similar to the main results, while the analysis for the females revealed no statistically significant associations. In the sensitivity analysis, increased sitting time was significantly associated with lower odds of having a high PA level, while education was not significantly associated anymore. The findings of our study might help to tailor future preventive strategies against NCD in Switzerland.

Our study showed that the PA level of the population of Switzerland was high; only 10% (individuals with low PA level) did not meet the minimum criteria of PA as defined by WHO [8]). Our results are consistent with the findings of the Swiss Health Survey in 2017, reporting that three out of four individuals were sufficiently physically active and only 9% were inactive [48]. According to Sport Switzerland and a study from Geneva, the levels of PA increased over the last years [49, 50]. In 2020, only 15% of the Swiss individuals were inactive, whereas over 30% were inactive in 1978 [50].

The combination of the Pulkstenis-Robinson and Hosmer-Lemeshow test suggested that the model fits the data reasonably well.

In our study, no evidence for an association of PA levels with sex was observed. Therefore, disparities in access to PA opportunities [51] and gender inequality, such as social expectations [52], leading to a lower PA level among females compared to males [23, 51–53], do not seem to be generally evident in Switzerland.

Compared to the reference age group (18–29), the two middle age groups (30–59) showed evidence for having lower odds of being in a higher PA level, whereas the oldest age group (60–76) did not. A potential explanation for the absence of an association could be the small sample size in the oldest and youngest age group compared to the two middle age groups, leading to wider confidence intervals. Additionally, the oldest age group probably included mostly retired participants, which have more free time they might have spent on physical activities, leading therefore to a statistically insignificant trend of lower odds to be in a higher PA level compared to the youngest age group. Meanwhile, in another Swiss study with participants over 52 years of age, it was observed that the PA level decreased with age [54]. We also found that individuals with a tertiary education were less physically active compared to individuals with a lower education. This contrasts with previous studies which indicate that various domains of PA – such as occupational PA, leisure-time PA, and transportational PA – exhibit distinct associations with educational status. Specifically, individuals with tertiary education tend to have higher levels of leisure-time PA, while those with lower education engage more in occupational PA [55–57]. This disparity highlights the importance of considering the specific domains in PA in future research to better understand and address inequalities in PA levels across different education levels.

In our study, individuals being obese and reporting medium to bad health status had lower odds of being in a higher PA level compared to the “respective” “reference” group, meanwhile the other lifestyle factors were not associated with PA levels. Previous studies have observed an association of high PA with normal BMI and a good general health status, whereas other studies also observed an association with non-smoking behavior [58–60]. The association between smoking and PA depends on the type of PA. Occupational or commuting PA are risk factors for smoking and leisure-time PA is associated with a lower smoking behavior [12]. PA also influences eating behavior, it has been shown that individuals engaging in regular PA tend to have a healthier diet [13, 14], however they tend to consume more alcohol [15–17]. We did not observe these associations in our analysis, potentially due to the small sample size and the cross-sectional study design, capturing data only at a single point in time. Additionally, factors such as smoking behavior were categorized relatively broadly (i.e. former, never, current), which could have limited the sensitivity of our analysis to detect associations.

To observe potential sex-based differences, we stratified our study population by sex. Statistically significant associations between PA levels and sociodemographic and lifestyle factors were observed for the male subgroup, whereas for the female subgroup similar trends were observed, which, however, were not statistically significant. Nonetheless, we suggest that the observed associations in the whole population and the male subgroup should also be considered when planning female-specific PA strategies. Further sex-specific studies with larger sample sizes are needed to assure our observed trends, especially among the female subgroup, are indeed significant associations with PA levels and therefore an effective target for future PA strategies.

As individuals with a tertiary education spend more time sitting, we conducted a sensitivity analysis and included sitting time in the model, which revealed that education was no longer statistically significantly associated with PA, while sitting time was. The latter findings suggest that the lower levels of PA of individuals with tertiary education were due to increased sitting time associated with education level rather than the education level *per se*. This finding highlighted the importance of including the sedentary behavior into analyses of PA behaviors. Research findings indicated that the relative risk for CVD linked to sedentary behavior is more pronounced among individuals with low levels of PA [61, 62]. Moreover, a sedentary behavior is a risk factor for CVD, independent of the PA level [63]. Notably, engaging in a high level of PA appears to mitigate the adverse consequences of sedentary behavior on cardiovascular health [64].

Our results should be considered with some limitation. In menuCH, PA and other variables (e.g. income, education level, health status, and smoking behavior) were collected via self-reporting, which can result in recall, information, and misclassification bias. PA was measured using the short IPAQ questionnaire, which does not distinguish between the different domains of PA, especially between leisure and occupation PA. PA data is missing for almost 25% of the participants, potentially introducing selection bias, as the characteristics of the individuals with missing PA may differ systematically from those who provided complete data. Lastly, menuCH is a cross-sectional study and therefore, no cause-effect conclusions can be drawn.

However, our study had important strengths. The menuCH study population is representative for the population of Switzerland due to the survey weighting strategy. Other strengths are the dietary assessment tool and the IPAQ. Two 24HDR were conducted and analyzed using the GloboDiet^®^ [38] by trained dietitians [36]. The IPAQ is an international and validated standard tool to measure PA. The last strength is the available data on sitting time, which could be included in the sensitivity analysis.

### Conclusion

The majority of the population of Switzerland met the WHO recommendations on PA. Sociodemographic and lifestyle factors, such as education level, age group, BMI group, and self-reported health status are related to an individual’s PA level. We observed that sedentary behavior is an important risk factor for lower PA levels. This is particularly concerning given the increasing sitting time observed in the population of Switzerland. Additionally, our findings highlight differences between males and females. Future studies should focus on sedentary behavior, with a clear distinction between occupational and leisure-time PA, as well as more detailed analysis to investigate the difference between sexes. The findings of our study might shape decisions of policymaker and thereby help to plan future preventive strategies against NCD.

## Data Availability

The data and further documents of the menuCH study are available by request under https://menuch.iumsp.ch.

## References

[1] WHO. Non-Communicable Diseases. Geneva, Switzerland: Key Facts (2023). Available from: https://www.who.int/news-room/fact-sheets/detail/noncommunicable-diseases (Accessed July 22, 2024).

[2] WHO. Monitoring Health for the SDGs: Sustainable Development Goals. Geneva (Switzerland): WHO (2018).

[3] FSO. The Impact of the COVID-19 Pandemic on Mortality and Causes of Death in Switzerland. Neuchâtel (2023).

[4] Chammartin F Probst-Hensch N Utzinger J Vounatsou P. Mortality Atlas of the Main Causes of Death in Switzerland, 2008-2012. Swiss Med Wkly (2016) 146:w14280. 10.4414/smw.2016.14280 26901250

[5] Martin-Diener E Meyer J Braun J Tarnutzer S Faeh D Rohrmann S The Combined Effect on Survival of Four Main Behavioural Risk Factors for Non-Communicable Diseases. Prev Med (Baltim) (2014) 65:148–52. 10.1016/j.ypmed.2014.05.023 24989976

[6] Loef M Walach H. The Combined Effects of Healthy Lifestyle Behaviors on All Cause Mortality: A Systematic Review and Meta-Analysis. Prev Med (Baltim) (2012) 55(3):163–70. 10.1016/j.ypmed.2012.06.017 22735042

[7] WHO. Non-Communicable Disease Country Profiles 2018. Geneva (Switzerland): WHO (2018).

[8] WHO. Global Recommendations on Physical Activity for Health. Geneva (Switzerland): WHO (2010). 26180873

[9] Schneider S Huy C Schuessler M Diehl K Schwarz S. Optimising Lifestyle Interventions: Identification of Health Behaviour Patterns by Cluster Analysis in a German 50+ Survey. Eur J Public Health (2009) 19(3):271–7. 10.1093/eurpub/ckn144 19164433

[10] Ware D Landy DC Rabil A Hennekens CH Hecht EM. Interrelationships Between Self Reported Physical Health and Health Behaviors Among Healthy US Adults: From the NHANES 2009–2016. Public Health Pract (2022) 4:100277. 10.1016/j.puhip.2022.100277 PMC9773046 36570399

[11] Tsai J Ford ES Li C Zhao G Pearson WS Balluz LS. Multiple Healthy Behaviors and Optimal Self-Rated Health: Findings From the 2007 Behavioral Risk Factor Surveillance System Survey. Prev Med (Baltim) (2010) 51(3–4):268–74. 10.1016/j.ypmed.2010.07.010 20647019

[12] Zhang J Cao Y Mo H Feng R. The Association Between Different Types of Physical Activity and Smoking Behavior. BMC Psychiatry (2023) 23(1):927. 10.1186/s12888-023-05416-1 38082223 PMC10712079

[13] Andrade AM Coutinho SR Silva MN Mata J Vieira PN Minderico CS The Effect of Physical Activity on Weight Loss Is Mediated by Eating Self-Regulation. Patient Educ Couns (2010) 79(3):320–6. 10.1016/j.pec.2010.01.006 20149955

[14] Loprinzi PD Smit E Mahoney S. Physical Activity and Dietary Behavior in US Adults and Their Combined Influence on Health. Mayo Clin Proc (2014) 89(2):190–8. 10.1016/j.mayocp.2013.09.018 24485132

[15] Courtney JB Russell MA Conroy DE. Tobacco and Cannabis Use as Moderators of the Association Between Physical Activity and Alcohol Use Across the Adult Lifespan in the United States: NHANES, 2005–2016. Prev Med (Baltim) (2022) 155:106931. 10.1016/j.ypmed.2021.106931 PMC8886825 34954238

[16] Leasure JL Neighbors C Henderson CE Young CM. Exercise and Alcohol Consumption: What We Know, What We Need to Know, and Why It Is Important. Front Psychiatry (2015) 6:156. 10.3389/fpsyt.2015.00156 26578988 PMC4629692

[17] Piazza-Gardner AK Barry AE. Examining Physical Activity Levels and Alcohol Consumption: Are People Who Drink More Active? Am J Health Prom (2012) 26(3):e95–104. 10.4278/ajhp.100929-LIT-328 22208422

[18] Lavie CJ Ozemek C Carbone S Katzmarzyk PT Blair SN. Sedentary Behavior, Exercise, and Cardiovascular Health. Circ Res (2019) 124(5):799–815. 10.1161/CIRCRESAHA.118.312669 30817262

[19] Piercy KL Troiano RP Ballard RM Carlson SA Fulton JE Galuska DA The Physical Activity Guidelines for Americans. JAMA (2018) 320:2020–8. 10.1001/jama.2018.14854 30418471 PMC9582631

[20] Bull FC Al-Ansari SS Biddle S Borodulin K Buman MP Cardon G World Health Organization 2020 Guidelines on Physical Activity and Sedentary Behaviour. Br J Sports Med (2020) 54(24):1451–62. 10.1136/bjsports-2020-102955 33239350 PMC7719906

[21] Ekelund U Tarp J Steene-Johannessen J Hansen BH Jefferis B Fagerland MW Dose-Response Associations Between Accelerometry Measured Physical Activity and Sedentary Time and All Cause Mortality: Systematic Review and Harmonised Meta-Analysis. The BMJ (2019) 366(l4570):l4570. 10.1136/bmj.l4570 31434697 PMC6699591

[22] Warburton DER Bredin SSD. Health Benefits of Physical Activity: A Systematic Review of Current Systematic Reviews. Curr Opin Cardiol (2017) 32(5):541–56. 10.1097/HCO.0000000000000437 28708630

[23] Hallal PC Andersen LB Bull FC Guthold R Haskell W Ekelund U Global Physical Activity Levels: Surveillance Progress, Pitfalls, and Prospects. The Lancet (2012) 380:247–57. 10.1016/S0140-6736(12)60646-1 22818937

[24] Lamprecht M Bürgi R Stamm H. Sport Schweiz Light 2022 Die Folgen der Covid-19-Pandemie für das Sportverhalten der Schweizer Bevölkerung Forschungsbericht (2022).

[25] Guthold R Stevens GA Riley LM Bull FC. Worldwide Trends in Insufficient Physical Activity From 2001 to 2016: A Pooled Analysis of 358 Population-Based Surveys with 1·9 Million Participants. Lancet Glob Health (2018) 6(10):e1077–86. 10.1016/S2214-109X(18)30357-7 30193830

[26] Guthold R Stevens GA Riley LM Bull FC. Global Trends in Insufficient Physical Activity Among Adolescents: A Pooled Analysis of 298 Population-Based Surveys With 1·6 Million Participants. Lancet Child Adolesc Health (2020) 4(1):23–35. 10.1016/S2352-4642(19)30323-2 31761562 PMC6919336

[27] Peng W Bai X Wu C Zhang H Li X Lu J. Sociodemographic Factors, Leisure-Time Physical Activity and Mortality. Am J Prev Med (2024) 66(4):598–608. 10.1016/j.amepre.2023.11.007 37972796

[28] Martland R Teasdale S Murray RM Gardner-Sood P Smith S Ismail K Dietary Intake, Physical Activity and Sedentary Behaviour Patterns in a Sample With Established Psychosis and Associations With Mental Health Symptomatology. Psychol Med (2023) 53(4):1565–75. 10.1017/S0033291721003147 34420532 PMC10009388

[29] Gottschlich EA Larson K Sisk B Pat Frintner M. Sleep, Physical Activity, and General Health Status: US Pediatricians and the General US Adult Population. Acad Pediatr (2019) 19(3):269–77. 10.1016/j.acap.2018.08.002 30103049

[30] Krieger JP Pestoni G Cabaset S Brombach C Sych J Schader C Cultural Differences in Diet and Determinants of Diet Quality in Switzerland: Results From the National Nutrition Survey menuCH. Nutrients (2019) 11(1):126. 10.3390/nu11010126 30634520 PMC6357532

[31] Chatelan A Beer-Borst S Randriamiharisoa A Pasquier J Blanco JM Siegenthaler S Major Differences in Diet across Three Linguistic Regions of Switzerland: Results From the First National Nutrition Survey menuCH. Nutrients (2017) 9(11):1163. 10.3390/nu9111163 29068399 PMC5707635

[32] Cuschieri S. The STROBE Guidelines. Saudi J Anaesth (2019) 13:S31–4. 10.4103/sja.SJA_543_18 30930717 PMC6398292

[33] Bochud M Beer-Borst S. Anthropometric Characteristics and Indicators of Eating and Physical Activity Behaviors in the Swiss Adult Population Results from menuCH 2014-2015. (2017).

[34] IPAQ. Guidelines for Data Processing and Analysis of the International Physical Activity Questionnaire (IPAQ)—Short Form (2005). Available from: https://www.ipaq.ki.se (Accessed May 9, 2024).

[35] Craig CL Marshall AL Sjöström M Bauman AE Booth ML Ainsworth BE International Physical Activity Questionnaire: 12-Country Reliability and Validity. Med Sci Sports Exerc (2003) 35(8):1381–95. 10.1249/01.MSS.0000078924.61453.FB 12900694

[36] Chatelan A Marques-Vidal P Bucher S Siegenthaler S Metzger N Zuberbuehler CA Lessons Learnt about Conducting a Multilingual Nutrition Survey in Switzerland: Results From menuCH Pilot Survey. Int J Vit Nutri Res (2017) 87(1–2):25–36. 10.1024/0300-9831/a000429 29676677

[37] Slimani N Casagrande C Nicolas G Freisling H Huybrechts I Ocké MC The Standardized Computerized 24-h Dietary Recall Method EPIC-Soft Adapted for Pan-European Dietary Monitoring. Eur J Clin Nutr (2011) 65:5–15. 10.1038/ejcn.2011.83 21731006

[38] Crispim SP De Vries JHM Geelen A Souverein OW Hulshof PJM Lafay L Two Non-Consecutive 24 H Recalls Using EPIC-Soft Software Are Sufficiently Valid for Comparing Protein and Potassium Intake Between Five European Centres-Results From the European Food Consumption Validation (EFCOVAL) Study. J Nutr (2011) 105(3):447–58. 10.1017/S0007114510003648 20875188

[39] Camenzind-Frey E Zuberbühler C. menuCH - Schweizerisches Fotobuch/Livre Photo Suisse/Manuale Fotografico Svizzero. Bern: Bundesamt für Gesundheit (BAG) und Bundesamt für Lebensmittelsicherheit und Veterinärwesen (BLV) (2014).

[40] EKAL. Orientierungshilfe Zum Alkoholkonsum (2018). Available from: https://www.bag.admin.ch/dam/bag/de/dokumente/npp/alkohol/ekal/orientierungshilfe-alkoholkonsum.pdf.download.pdf/2-d-2015-orientierungshilfe-langversion.pdf (Accessed July 22, 2024).

[41] Bae D Wróbel A Kaelin I Pestoni G Rohrmann S Sych J. Investigation of Alcohol-Drinking Levels in the Swiss Population: Differences in Diet and Associations With Sociodemographic, Lifestyle and Anthropometric Factors. Nutrients (2022) 14:2494. 10.3390/nu14122494 35745224 PMC9230148

[42] Pestoni G Krieger JP Sych JM Faeh D Rohrmann S. Cultural Differences in Diet and Determinants of Diet Quality in switzerland: Results From the National Nutrition Survey menuCH. Nutrients (2019) 11(1):126. 10.3390/nu11010126 30634520 PMC6357532

[43] Pasquier J Chatelan A Bochud M. Weighting Strategy (2017). Available from: https://www.data.blv.admin.ch/catalog/4#metadata-sampling (Accessed May 9, 2024).

[44] Azur MJ Stuart EA Frangakis C Leaf PJ. Multiple Imputation by Chained Equations: What Is It and How Does It Work? Int J Methods Psychiatr Res (2011) 20(1):40–9. 10.1002/mpr.329 21499542 PMC3074241

[45] Royston P. Multiple Imputation of Missing Values. Stata J (2004) 4(3):227–41. 10.1177/1536867x0400400301

[46] Fagerland MW Hosmer DW. Tests for Goodness of Fit in Ordinal Logistic Regression Models. J Stat Comput Simul (2016) 86(17):3398–418. 10.1080/00949655.2016.1156682

[47] R core Team. The R Project for Statistical Computing (2021). Available from: https://www.r-project.org/ (Accessed June 24, 2024).

[48] BFS. Schweizerische Gesundheitsbefragung 2017. Neuchâtel (2017).

[49] Guessous I Gaspoz JM Theler JM Kayser B. Eleven-Year Physical Activity Trends in a Swiss Urban Area. Prev Med (Baltim) (2014) 59(1):25–30. 10.1016/j.ypmed.2013.11.005 24252488

[50] Lamprecht M Bürgi R Stamm H für Sport Baspo B. Sport Schweiz 2020 Sportaktivität und Sportinteresse der Schweizer Bevölkerung. Zürich (2020).

[51] Brown WJ Mielke GI Kolbe-Alexander TL. Gender Equality in Sport for Improved Public Health. The Lancet (2016) 388(10051):1257–8. 10.1016/S0140-6736(16)30881-9 27475268

[52] Moreno-Llamas A García-Mayor J De la Cruz-Sánchez E. Gender Inequality Is Associated With Gender Differences and Women Participation in Physical Activity. J Public Health (Bangkok) (2022) 44(4):e519–26. 10.1093/pubmed/fdab354 34604911

[53] Althoff T Sosič R Hicks JL King AC Delp SL Leskovec J. Large-scale Physical Activity Data Reveal Worldwide Activity Inequality. Nature (2017) 547(7663):336–9. 10.1038/nature23018 28693034 PMC5774986

[54] Aebi NJ Bringolf-Isler B Schaffner E Caviezel S Imboden M Probst-Hensch N. Patterns of Cross-Sectional and Predictive Physical Activity in Swiss Adults Aged 52+: Results From the SAPALDIA Cohort. Swiss Med Wkly (2020) 150(25–26):w20266. 10.4414/smw.2020.20266 32579698

[55] O’Donoghue G Kennedy A Puggina A Aleksovska K Buck C Burns C Socio-Economic Determinants of Physical Activity Across the Life Course: A “DEterminants of DIet and Physical ACtivity” (DEDIPAC) Umbrella Literature Review. PLoS One (2018) 13(1):e0190737. 10.1371/journal.pone.0190737 29351286 PMC5774703

[56] Bauman AE Reis RS Sallis JF Wells JC Loos RJF Martin BW Correlates of Physical Activity: Why Are Some People Physically Active and Others Not? The Lancet (2012) 380(9838):258–71. 10.1016/S0140-6736(12)60735-1 22818938

[57] Beenackers MA Kamphuis CB Giskes K Brug J Kunst AE Burdorf A Socioeconomic Inequalities in Occupational, Leisure-Time, and Transport Related Physical Activity Among European Adults: A Systematic Review. Int J Behav Nutri Phys Act (2012) 9(116):116. 10.1186/1479-5868-9-116 PMC3491027 22992350

[58] Södergren M Wang WC Salmon J Ball K Crawford D McNaughton SA. Predicting Healthy Lifestyle Patterns Among Retirement Age Older Adults in the WELL Study: A Latent Class Analysis of Sex Differences. Maturitas (2014) 77(1):41–6. 10.1016/j.maturitas.2013.09.010 24144958

[59] Lisha NE Sussman S. Relationship of High School and College Sports Participation With Alcohol, Tobacco, and Illicit Drug Use: A Review. Addict Behav (2010) 35(5):399–407. 10.1016/j.addbeh.2009.12.032 20100638 PMC3134407

[60] Schuit AJ Van Loon AJM Tijhuis M Ocké MC. Clustering of Lifestyle Risk Factors in a General Adult Population. Prev Med (Baltim) (2002) 35(3):219–24. 10.1006/pmed.2002.1064 12202063

[61] Katzmarzyk PT Ross R Blair SN Després JP. Should We Target Increased Physical Activity or Less Sedentary Behavior in the Battle against Cardiovascular Disease Risk Development? Atherosclerosis (2020) 311:107–15. 10.1016/j.atherosclerosis.2020.07.010 32773106

[62] Liang ZD Zhang M Wang CZ Yuan Y Liang JH. Association Between Sedentary Behavior, Physical Activity, and Cardiovascular Disease-Related Outcomes in Adults—A Meta-Analysis and Systematic Review. Front Public Health (2022) 10:1018460–15. 10.3389/fpubh.2022.1018460 36339165 PMC9632849

[63] Patterson R McNamara E Tainio M Hérick De Sá T Smith AD Sharp SJ Sedentary Behaviour and Risk of All-Cause, Cardiovascular and Cancer Mortality, and Incident Type 2 Diabetes: A Systematic Review and Dose Response Meta-Analysis. Eur J Epidemiol (2018) 33:811–29. 10.1007/s10654-018-0380-1 29589226 PMC6133005

[64] Gibbs BB Hergenroeder AL Katzmarzyk PT Lee IM Jakicic JM. Definition, Measurement, and Health Risks Associated with Sedentary Behavior. In: Med Sci Sports Exerc. Lippincott Williams and Wilkins (2015). p. 1295–300. 10.1249/MSS.0000000000000517 PMC4362881 25222816

