# Supplementary material for: Investigation of Physical Activity Levels in the Population of Switzerland: Association With Lifestyle and Sociodemographic Factors
Source: Int J Public Health. 2025 Mar 11;70:1608010. doi: 10.3389/ijph.2025.1608010 (PMC11932833; doi:10.3389/ijph.2025.1608010)
Supplement: Supplementary file 1 [file DataSheet1.docx]

## Supplemental Information

Table SI 1 Results of ordinal logistic regression, stratified by sex. (National Nutrition Survey menuCH, Switzerland, 2014-2015, n = 2057)

|  | **OR^a^** | **95 % CI** | **OR^a^** | **95 % CI** |
| --- | --- | --- | --- | --- |
|  | **Females** | | **Males** | |
| **BMI** | |  |  |  |
| Normal (ref.) | 1.00 | - | 1.00 | - |
| Underweight | 1.23 | [0.59, 2.60] | 0.86 | [0.10, 7.11] |
| Overweight | 0.79 | [0.56, 1.11] | 0.90 | [0.67, 1.22] |
| Obese | 0.64 | [0.39, 1.06] | 0.67 | [0.41, 1.10] |
| **Age** | |  |  |  |
| 18 – 29 (ref.) | 1.00 | - | 1.00 | - |
| 30 – 44 | 0.66 | [0.43, 1.02] | 0.38 | [0.22, 0.68]* |
| 45 – 59 | 0.75 | [0.45, 1.23] | 0.43 | [0.23, 0.79]* |
| 60 – 76 | 1.01 | [0.57, 1.78] | 0.67 | [0.36, 1.27] |
| **Language region** | | |  |  |
| German (ref.) | 1.00 | - | 1.00 | - |
| French | 0.95 | [0.68, 1.32] | 1.42 | [1.00, 2.01] |
| Italian | 0.81 | [0.45, 1.46] | 0.89 | [0.49, 1.62] |
| **Nationality** | |  |  |  |
| Swiss (ref.) | 1.00 | - | 1.00 | - |
| Non-Swiss | 0.91 | [0.62, 1.33] | 0.73 | [0.49, 1.11] |
| Swiss binationals | 1.07 | [0.71, 1.62] | 0.92 | [0.60, 1.42] |
| **Education** | |  |  |  |
| Primary (ref.) | 1.00 | - | 1.00 | - |
| Secondary | 0.72 | [0.32, 1.66] | 0.46 | [0.21, 0.99]* |
| Tertiary | 0.55 | [0.23, 1.28] | 0.45 | [0.42, 0.95]* |
| **Health status** | |  |  |  |
| Good (ref.) | 1.00 | - | 1.00 | - |
| Medium-bad | 0.82 | [0.51, 1.31] | 0.63 | [0.42, 0.95]* |
| **Smoking** | |  |  |  |
| Never (ref.) | 1.00 | - | 1.00 | - |
| Former | 1.16 | [0.87, 1.55] | 0.89 | [0.62, 1.28] |
| Current | 1.35 | [0.89, 2.03] | 0.87 | [0.59, 1.29] |
| **Civil status** | |  |  |  |
| Single (ref.) | 1.00 | - | 1.00 | - |
| Married | 1.10 | [0.75, 1.62] | 1.06 | [0.70, 1.61] |
| Divorced | 1.23 | [0.74, 2.04] | 1.38 | [0.76, 2.51] |
| Other | 0.66 | [0.35, 1.22] | 1.50 | [0.49, 4.55] |
| **Currently on a diet** | |  |  |  |
| No (ref.) | 1.00 | - | 1.00 | - |
| Yes | 0.92 | [0.53, 1.61] | 0.82 | [0.37, 1.81] |
| **Alcohol intake** | | |  |  |
| None (ref.) | 1.00 | - | 1.00 | - |
| Moderate | 0.96 | [0.68, 1.34] | 1.03 | [0.70, 1.50] |
| Heavy | 0.88 | [0.60, 1.28] | 1.15 | [0.79, 1.66] |
| **AHEI score** | 1.01 | [0.99, 1.02] | 0.99 | [0.98, 1.01] |

*p < .05

BMI body mass index, AHEI alternative healthy eating index, OR odds ratio, CI confidence interval

^a^ The OR are weighted according to the menuCH weighting strategy for sex, age, marital status, major living region Switzerland, nationality, household size, weekday and season of the recall day.^1^

Table SI 2 Results of the sensitivity analysis for sociodemographic and lifestyle factors associated with physical activity level, including income as additional explanatory variable. (National Nutrition Survey menuCH, Switzerland, 2014-2015, n = 2057)

|  | **OR^a^** | **95 % CI** |
| --- | --- | --- |
| **Sex** |  |  |
| Male (ref.) | 1.00 | - |
| Female | 0.93 | [0.76, 1.15] |
| **BMI** | |  |
| Normal (ref.) | 1.00 | - |
| Underweight | 1.16 | [0.58, 2.31] |
| Overweight | 0.84 | [0.67, 1.05] |
| Obese | 0.63 | [0.45, 0.90]* |
| **Age** | |  |
| 18 – 29 (ref.) | 1.00 | - |
| 30 – 44 | 0.54 | [0.38, 0.78]* |
| 45 – 59 | 0.61 | [0.42, 0.90]* |
| 60 – 76 | 0.88 | [0.59, 1.30] |
| **Language region** | | |
| German (ref.) | 1.00 | - |
| French | 1.12 | [0.89, 1.42] |
| Italian | 0.85 | [0.56, 1.29] |
| **Nationality** | |  |
| Swiss (ref.) | 1.00 | - |
| Non-Swiss | 0.80 | [0.60, 1.07] |
| Swiss binationals | 0.98 | [0.73, 1.32] |
| **Education** | |  |
| Primary (ref.) | 1.00 | - |
| Secondary | 0.59 | [0.34, 1.05] |
| Tertiary | 0.51 | [0.29, 0.90\|* |
| **Health status** | |  |
| Good (ref.) | 1.00 | - |
| Medium-bad | 0.68 | [0.50, 0.92]* |
| **Smoking** | |  |
| Never (ref.) | 1.00 | - |
| Former | 1.01 | [0.81, 1.26] |
| Current | 1.01 | [0.77, 1.32] |
| **Civil status** | |  |
| Single (ref.) | 1.00 | - |
| Married | 1.07 | [0.82, 1.41] |
| Divorced | 1.23 | [0.84, 1.81] |
| Other | 0.86 | [0.56, 1.53] |
| **Currently on a diet** | |  |
| No (ref.) | 1.00 | - |
| Yes | 0.88 | [0.61, 1.37] |
| **Alcohol intake** | | |
| None (ref.) | 1.00 | - |
| Moderate | 0.96 | [0.75, 1.24] |
| Heavy | 1.00 | [0.78, 1.30] |
| **Income (CHF/month)** | |  |
| <6000 (ref.) | 1.00 | - |
| 6000 - 13000 | 0.85 | [0.61, 1.18] |
| >13000 | 0.93 | [0.64, 1.37] |
| **AHEI score** | 1.00 | [0.99, 1.01] |

*p < .05

BMI body mass index, AHEI alternative healthy eating index, CHF = Swiss francs, OR odds ratio, CI confidence interval

^a^ The OR are weighted according to the menuCH weighting strategy for sex, age, marital status, major living region Switzerland, nationality, household size, weekday and season of the recall day.^1^

Table SI 3 Results of the sensitivity analysis for sociodemographic and lifestyle factors associated with physical activity level, including sitting time as additional explanatory variable. (National Nutrition Survey menuCH, Switzerland, 2014-2015, n = 2057)

|  | | **OR^a^** | **95 % CI** |
| --- | --- | --- | --- |
| **Sex** | |  |  |
| Male (ref.) | | 1.00 | - |
| Female | | 0.87 | [0.70, 1.07] |
| **BMI** | | |  |
| Normal (ref.) | | 1.00 | - |
| Underweight | | 1.28 | [0.62, 2.65] |
| Overweight | | 0.84 | [0.66, 1.06] |
| Obese | | 0.66 | [0.47, 0.93]* |
| **Age** | | |  |
| 18 – 29 (ref.) | | 1.00 | - |
| 30 – 44 | | 0.50 | [0.35, 0.73]* |
| 45 – 59 | | 0.61 | [0.41, 0.90]* |
| 60 – 76 | | 0.77 | [0.51, 1.16] |
| **Language region** | | | |
| German (ref.) | | 1.00 | - |
| French | | 1.03 | [0.80, 1.32] |
| Italian | | 0.72 | [0.47, 1.09] |
| **Nationality** | | |  |
| Swiss (ref.) | | 1.00 | - |
| Non-Swiss | | 0.85 | [0.63, 1.15] |
| Swiss binationals | | 1.03 | [0.77, 1.38] |
| **Education** | | |  |
| Primary (ref.) | 1.00 | | - |
| Secondary | | 0.64 | [0.37, 1.12] |
| Tertiary | | 0.66 | [0.38, 1.13\| |
| **Health status** | | |  |
| Good (ref.) | | 1.00 | - |
| Medium-bad | | 0.68 | [0.50, 0.92]* |
| **Smoking** | | |  |
| Never (ref.) | | 1.00 | - |
| Former | | 1.01 | [0.80, 1.27] |
| Current | | 0.96 | [0.73, 1.26] |
| **Civil status** | | |  |
| Single (ref.) | | 1.00 | - |
| Married | | 0.89 | [0.67, 1.18] |
| Divorced | | 1.02 | [0.69, 1.50] |
| Other | | 0.69 | [0.38, 1.23] |
| **Currently on a diet** | | |  |
| No (ref.) | | 1.00 | - |
| Yes | | 0.95 | [0.61, 1.48] |
| **Alcohol Intake** | | | |
| None (ref.) | | 1.00 | - |
| Moderate | | 0.97 | [0.75, 1.25] |
| Heavy | | 1.05 | [0.80, 1.36] |
| **AHEI score** | | 1.00 | [0.99, 1.01] |
| **Sitting time (h)** | | 0.88 | [0.85, 0.91]* |

*p < .05

BMI body mass index, AHEI alternative healthy eating index, OR odds ratio, CI confidence interval, h hours

^a^ The OR are weighted according to the menuCH weighting strategy for sex, age, marital status, major living region Switzerland, nationality, household size, weekday and season of the recall day.^1^

Table SI 4 Results of the sensitivity analysis for sociodemographic and lifestyle factors associated with physical activity level, including income and sitting time as additional explanatory variables. (National Nutrition Survey menuCH, Switzerland, 2014-2015, n = 2057)

|  | **OR^a^** | **95% CI** |
| --- | --- | --- |
| **Sex** |  |  |
| Male (ref.) | 1.00 | - |
| Female | 0.87 | [0.70, 1.07] |
| **BMI** | |  |
| Normal (ref.) | 1.00 | - |
| Underweight | 1.30 | [0.63, 2.70] |
| Overweight | 0.84 | [0.66, 1.06] |
| Obese | 0.66 | [0.47, 0.93]* |
| **Age** | |  |
| 18 – 29 (ref.) | 1.00 | - |
| 30 – 44 | 0.51 | [0.35, 0.74]* |
| 45 – 59 | 0.60 | [0.41, 0.89]* |
| 60 – 76 | 0.78 | [0.51, 1.17] |
| **Language region** | | |
| German (ref.) | 1.00 | - |
| French | 1.03 | [0.81, 1.33] |
| Italian | 0.73 | [0.48, 1.11] |
| **Nationality** | |  |
| Swiss (ref.) | 1.00 | - |
| Non-Swiss | 0.85 | [0.63, 1.14] |
| Swiss binationals | 1.02 | [0.75, 1.36] |
| **Education** | |  |
| Primary (ref.) | 1.00 | - |
| Secondary | 0.65 | [0.37, 1.14] |
| Tertiary | 0.66 | [0.38, 1.14] |
| **Health status** | |  |
| Good (ref.) | 1.00 | - |
| Medium-bad | 0.68 | [0.50, 0.99]* |
| **Smoking** | |  |
| Never (ref.) | 1.00 | - |
| Former | 1.01 | [0.81, 1.27] |
| Current | 0.96 | [0.73, 1.26] |
| **Civil status** | |  |
| Single (ref.) | 1.00 | - |
| Married | 0.89 | [0.66, 1.19] |
| Divorced | 1.02 | [0.69, 1.52] |
| Other | 0.66 | [0.37, 1.19] |
| **Currently on a diet** | |  |
| No (ref.) | 1.00 | - |
| Yes | 0.95 | [0.61, 1.49] |
| **Alcohol intake** | | |
| None (ref.) | 1.00 | - |
| Moderate | 0.96 | [0.75, 1.24] |
| Heavy | 1.04 | [0.79, 1.36] |
| **Income** | |  |
| < 6000 CHF/month (ref.) | 1.00 | - |
| 6000 - 13000 CHF/month | 0.95 | [0.67, 1.33] |
| >13000 CHF/month | 1.15 | [0.78, 1.68] |
| **AHEI score** | 1.00 | [0.99, 1.01] |
| **Sitting time (h)** | 0.88 | [0.84, 0.91]* |

*p < .05

BMI body mass index, AHEI alternative healthy eating index, CHF = Swiss francs, OR odds ratio, CI confidence interval, h = hours

^a^ The OR are weighted according to the menuCH weighting strategy for sex, age, marital status, major living region Switzerland, nationality, household size, weekday and season of the recall day.^1^

## References

1. Pasquier J, Chatelan A, Bochud M. Weighting strategy. https://www.data.blv.admin.ch/catalog/4#metadata-sampling. Published 2017. Accessed May 9, 2024.
